# Spectris™ treatment preserves corpus callosum structure in Alzheimer's disease

**DOI:** 10.3389/fneur.2024.1452930

**Published:** 2024-10-15

**Authors:** Xiao Da, Evan Hempel, Adam M. Brickman, Mihály Hajós, Ralph Kern, Aylin Cimenser

**Affiliations:** ^1^Cognito Therapeutics, Inc, Cambridge, MA, United States; ^2^Taub Institute for Research on Alzheimer's Disease and the Aging Brain, Vagelos College of Physicians and Surgeons, Columbia University, New York, NY, United States; ^3^Department of Neurology, Vagelos College of Physicians and Surgeons, Columbia University, New York, NY, United States; ^4^Department of Comparative Medicine, Yale University School of Medicine, New Haven, CT, United States

**Keywords:** Alzheimer's disease, corpus callosum, white matter, atrophy, 40Hz, non-invasive, gamma, stimulation

## Abstract

**Objective:**

To examine the impact of 40Hz gamma stimulation on the preservation of the corpus callosum, a critical structure for interhemispheric connectivity, in people with mild cognitive impairment or Alzheimer's disease.

**Methods:**

OVERTURE (NCT03556280) participants were randomized 2:1 (Active:Sham) to receive daily, 1-h, 40Hz gamma sensory stimulation or sham treatment for 6 months. Structural magnetic resonance imaging data were analyzed to assess changes in corpus callosum area (*N* = 50; 33 for active, 17 for sham). Bayesian linear mixed-effects modeling was used to assess differences in longitudinal changes of corpus callosum area between the two treatment groups.

**Results:**

All observed differences in corpus callosum area favored the active treatment group. Differences were observed in the total corpus callosum area (2.28 ± 0.87%, *p* < 0.02) and its subregions, including genu/rostrum (2.36 ± 0.90%, *p* < 0.02), anterior-body (2.64 ± 1.26%, *p* < 0.04), mid-body (2.79 ± 1.18%, *p* < 0.03), posterior-body (2.87 ± 1.41%, *p* < 0.05), and splenium (1.58 ± 0.73%, *p* < 0.04). Total corpus callosum area and some of the sub-regional differences, such as genu/rostrum and splenium, were observed as early as 3 months after commencement of treatment.

**Interpretation:**

The structural magnetic resonance imaging results from the OVERTURE Phase 2 study suggest that 6 months of non-invasive 40Hz stimulation reduces the rate of atrophy of the corpus callosum in individuals with Alzheimer's disease. The preservation of structural integrity in the corpus callosum, crucial for interhemispheric communication and cognitive function, may be achievable through this non-invasive approach, potentially providing a promising disease-modifying alternative in Alzheimer's disease management.

## Introduction

Alzheimer's disease (AD) is marked by neuronal death, synaptic loss, and compromised white matter structural integrity (1). Current AD therapies primarily focus on reducing amyloid protein accumulation, with limited focus on directly preventing neurodegeneration. Our prior research indicated that 40Hz gamma sensory stimulation may prevent atrophy in individuals with mild cognitive impairment (MCI) or AD, showing reductions in white matter volume loss (2, 3). However, our previous work did not specifically examine the corpus callosum. Notably, earlier studies, some over two decades old, reported more than eightfold reduction in the midsagittal area of the corpus callosum, the principal white matter fiber bundle connecting the brain's hemispheres, in AD patients compared to controls (4). Building on these findings, this study explored the effects of 40Hz gamma sensory stimulation therapy specifically on the preservation of the corpus callosum.

The corpus callosum plays a crucial role in interhemispheric functional integration, with over 300 million fibers transmitting information across the network (5). Corpus callosum atrophy is particularly pronounced in AD and preventing it may have important consequences for neuronal communication and neural network function (4, 6). Corpus callosum atrophy is more severe in the anterior and posterior thirds, where there are more small, myelinated fibers (7). Although the regional pattern and degree of corpus callosum atrophy correlates with dementia severity in individuals with AD (4, 8–12), the development of therapeutic interventions for AD have not focused on preventing or mitigating corpus callosum atrophy. Recent findings indicate that noninvasive 40Hz gamma sensory stimulation can induce neuronal oscillations, potentially preventing synaptic loss, neurodegeneration, and brain atrophy (13, 14). This stimulation may also mitigate demyelination and promote the generation of new oligodendrocytes (15). Furthermore, clinical studies suggest that 40Hz gamma stimulation may enhance functional connectivity and reduce potentially harmful cytokines in the nervous system (16), slow cognitive decline (2), and improve activities of daily living (2, 17).

## Materials and methods

### Study sample and design

This study used data from the OVERTURE clinical trial (NCT03556280), a randomized, placebo-controlled study aimed at evaluating the safety, tolerability, adherence, and efficacy estimate of daily, 1-h sessions using Spectris™ to generate combined 40Hz visual and auditory evoked gamma oscillations for 6 months in individuals who are clinically diagnosed as MCI or AD (*N* = 74) (2). The trial protocols were approved by the Advarra institutional review board (FDA IORG#0000635, OHRA IRB Registration #00000971), and informed consent was obtained from all participants or their legally authorized representatives. The exclusion criteria included comorbid neurological conditions, profound sensory impairments, seizure history, or those on anti-seizure/anti-epileptic medication. Participants who were already taking cholinesterase inhibitors continued a consistent dosage; however, the use of memantine was exclusionary. Although not part of the inclusion criteria, amyloid pathology was assessed using Amyvid [^18^F] florbetapir PET imaging [see Hajós et al. (2) for methods].

### Therapeutic device

The treatment used the Cognito Therapeutics device (Spectris™), featuring a handheld controller, visual stimulation eye-set, and headphones for auditory stimulation. The device allowed participants to adjust the visual and auditory stimulation levels for comfort and was equipped with a communication feature for assistance from a care partner if required. Usage and adherence data were automatically captured and transmitted to a secure cloud server for remote monitoring purposes.

### MRI data acquisition

Structural magnetic resonance imaging (MRI) was performed at baseline, month 3, and month 6 using 1.5 Tesla MRI scanners across multiple sites. The imaging protocol was harmonized with the Alzheimer's Disease Neuroimaging Initiative 1 (ADNI1) protocol to ensure consistency in data acquisition (3, 18). The protocol specifics for T1-weighted images were as follows: on Siemens Espree scanners, images were acquired with an in-plane spatial resolution of 1.25 × 1.25 mm, slice thickness of 1.2 mm, repetition time (TR) of 2,400 ms, and echo time (TE) of 3.65ms; on General Electric Signa HDxt scanners, the resolution was 0.94 × 0.94 mm, slice thickness 1.2 mm, TR ~3.9 ms, and TE 1.35 ms; and on Philips Ingenia or Achieva scanners, the protocol was set to 0.94 × 0.94 mm resolution, 1.2-mm slice thickness, TR of 9.5 ms, and TE ~3.6 or 4 ms.

### MRI analysis

Among the 74 participants who enrolled in the study, MRI analyses were conducted based on specific exclusion criteria to ensure the quality and reliability of the data. The exclusion criteria were as follows: Insufficient Image Quality: Participants whose T1-weighted (T1w) MRI images were deemed inadequate for analysis due to excessive motion artifacts or insufficient contrast between gray matter and white matter were also excluded. This criterion resulted in the exclusion of ten participants from the Active group and seven participants from the Sham group. Incomplete Data: Participants who failed to complete baseline structural MRI assessments or at least one follow-up visit by the end of the study period (Month 6) were excluded. This criterion led to the exclusion of three participants from the Active group and four participants from the Sham group. After applying these exclusion criteria, the final sample comprised 50 participants, with 33 in the Active group and 17 in the Sham group (see Table 1 for a comparison of the baseline characteristics of the Active and Sham groups in this sample). These participants were included in the longitudinal area assessments of the corpus callosum.

**Table 1:** Demographic characteristics^*^.

	**Active (*n* = 33)**	**Sham (*n* = 17)**	**χ^2^**	***t*-value**	**95% CI**	***p*-value**
Age in years, mean ± SD	69.21 ± 7.54	75.06 ± 11.26	n.a.	1.93	−0.41, 12.11	0.07
Sex, male/female	10/23	10/7	2.71	n.a.	n.a.	0.10
Number (%) of APOE^¶^ε4 positive	16 (50%)	8 (47%)	0	n.a.	n.a.	1
Number (%) of PET SUVR*^§^*>1.12	23 (77%)	11 (65%)	0.78	n.a.	n.a.	0.5
MMSE^†^ score, mean ± SD	21.15 ± 3.45	18.88 ± 3.33	n.a.	−2.25	−4.32, −0.22	0.03
ADCS-ADL^‡^ scale, mean ± SD	65.30 ± 9.47	62.94 ± 11.77	n.a.	−0.72	−9.12, 4.41	0.48

^*^From participants with corpus callosum data (see Material and methods).

^¶^APOE, apolipoprotein E. APOE status for one active participant is missing.

^§^PET SUVR (F18-AV-45 Positron Emission Tomography, Standard Uptake Value Ratio, whole cerebellum reference) (2). PET SUVR for three active treatment participants is missing.

^†^MMSE, Mini-Mental State Examination.

^‡^ADCS-ADL, Alzheimer's Disease Cooperative Study-Activities of Daily Living.

n.a., not applicable.

### Corpus callosum midsagittal area analysis

To delineate midsagittal area of the corpus callosum, we used a segmentation process using the Yuki module within the Automated Registration Toolbox (ART) (19) (http://www.nitrc.org/projects/art), which was applied to T1-weighted MRI images to define the boundaries and calculate the area within the midsagittal plane (MSP). Subsequently, the MSP of the corpus callosum was parcellated into five subregions (genu/rostrum, anterior body, mid body, posterior body, and splenium) (6) using an automated image analysis streamline. For baseline comparisons between the two groups, the area of interest for each individual was normalized by the total intracranial volume raised to the two-thirds power to account for the dimensional difference between volume and area.

### Statistical methods

A longitudinal analysis was conducted to assess percent changes in midsagittal plane corpus callosum area and in the areas of its five midsagittal plane subregions. The percent change was computed using the formula: *Percent change* = *100* × *(Area at follow-up/Area at baseline – 1) %* where *Area* represents the studied corpus callosum area at the respective time points. These changes were analyzed with a Bayesian linear mixed-effects model, incorporating non-informative priors for all model parameters. The model accounted for baseline Mini-Mental State Examination (MMSE) score, age at baseline, time of visit, treatment group allocation, baseline MRI measures of corpus callosum area, two-thirds power of total intracranial volume (as a covariate for area), group and visit interaction, and interactions between baseline area and visit. Random effects in the model included inter-subject and inter-site variability. The Kenward-Roger approximation was used to estimate the model's degrees of freedom. The percent change from baseline was evaluated for the total corpus callosum and each of the five subregions. All statistical analyses were performed using the statistical computing software R, version 4.1.1.

## Results

### Demographic characteristics

There were no differences in age, sex, apolipoprotein E4 (APOE4) status, or baseline Alzheimer's Disease Cooperative Study-Activities of Daily Living (ADCS-ADL) scores between active and sham groups (Table 1). However, MMSE scores were higher in the active group. This difference was accounted for and adjusted in all the models studied.

### Assessment of corpus callosum structure at baseline

The two treatment groups did not differ in total or subregion corpus callosum area at baseline (Table 2).

**Table 2:** Normalized area assessments for corpus callosum and its subregions at baseline.

	**Active (*n* = 33) mean ±SD (%)**	**Sham (*n* = 17) mean ±SD (%)**	***t*-value**	**95% CI**	***p*-value**
Total corpus callosum	4.17 ± 0.52	3.91 ± 0.55	−1.66	−0.59 to 0.06	0.108
Genu/rostrum	1.19 ± 0.15	1.14 ± 0.18	−0.98	−0.16 to 0.05	0.334
Anterior-body	0.61 ± 0.11	0.56 ± 0.09	−1.58	−0.10 to 0.01	0.123
Mid-body	0.55 ± 0.09	0.50 ± 0.08	−1.95	−0.10 to 0.00	0.059
Posterior-body	0.57 ± 0.12	0.52 ± 0.11	−1.22	−0.11 to 0.03	0.230
Splenium	1.26 ± 0.19	1.18 ± 0.16	−1.56	−0.18 to 0.02	0.127

### Changes in total corpus callosum following treatment

**Figure 1:**
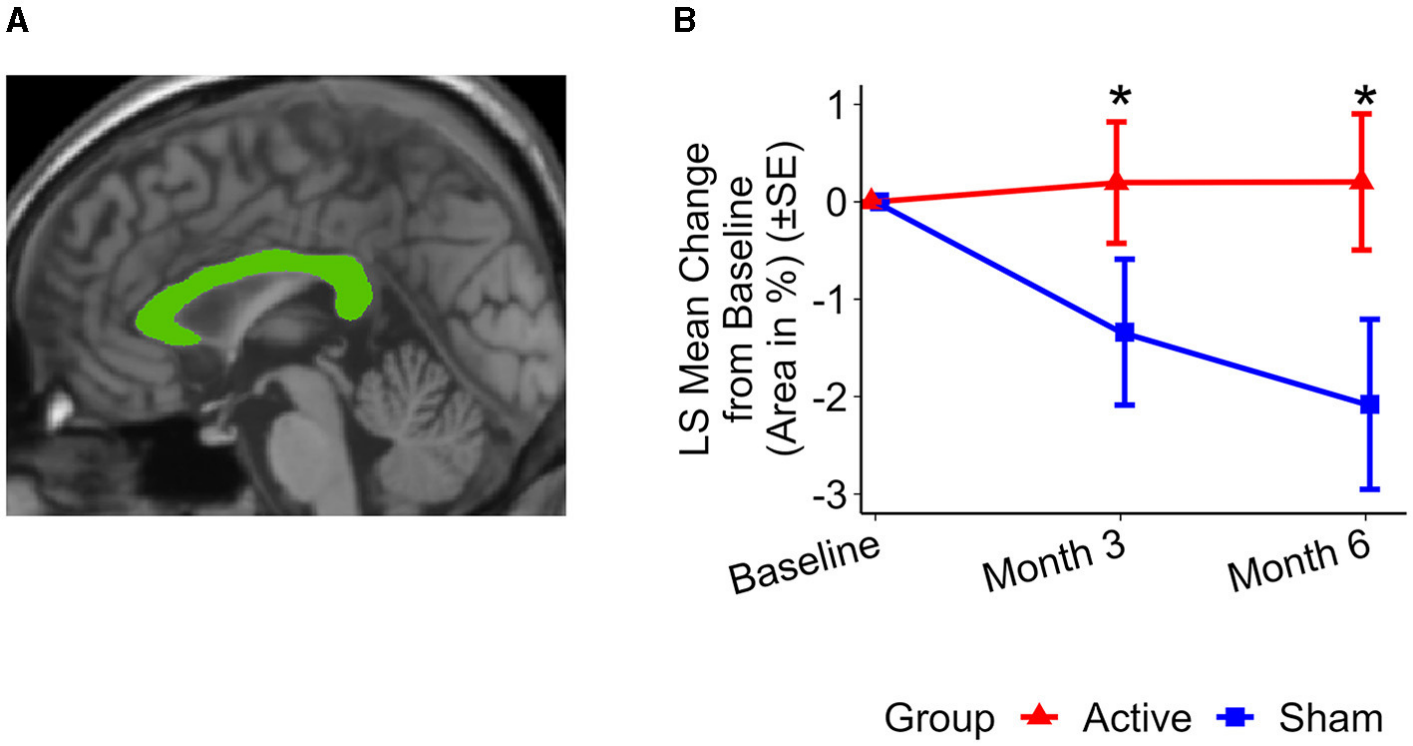
Representative total MSP corpus callosum area and group level changes (%) from baseline to 6 months. **(A)** Total MSP corpus callosum segmentation (green) from a single participant. **(B)** Least squares (LS) mean changes in total corpus callosum area. Significant differences were observed in the changes in total corpus callosum area between the active treatment group (red) and the sham group (blue) over both a 3-month period (*p* < 0.03) and a 6-month period (*p* < 0.02). The active treatment group showed preservation of the corpus callosum area, while the sham group exhibited atrophy. Error bars indicate standard error (SE). ^*^p < 0.05.

The active treatment group exhibited preservation of the corpus callosum, in contrast to the sham group, which demonstrated atrophy (see Figure 1): The differences were detectable after 3 months; the active treatment group had a change of 0.20 ± 0.62% while the sham group had a change of −1.34 ± 0.75%. The difference between the groups was significant after 3 months (1.54 ± 0.68%, *p* < 0.03). After 6 months of treatment, the active group exhibited a corpus callosum area change of 0.20 ± 0.70%, whereas the sham group showed a change of −2.08 ± 0.87%. The difference between the two groups was significant (2.28 ± 0.87%, *p* < 0.02).

### Changes in corpus callosum subregional areas

**Figure 2:**
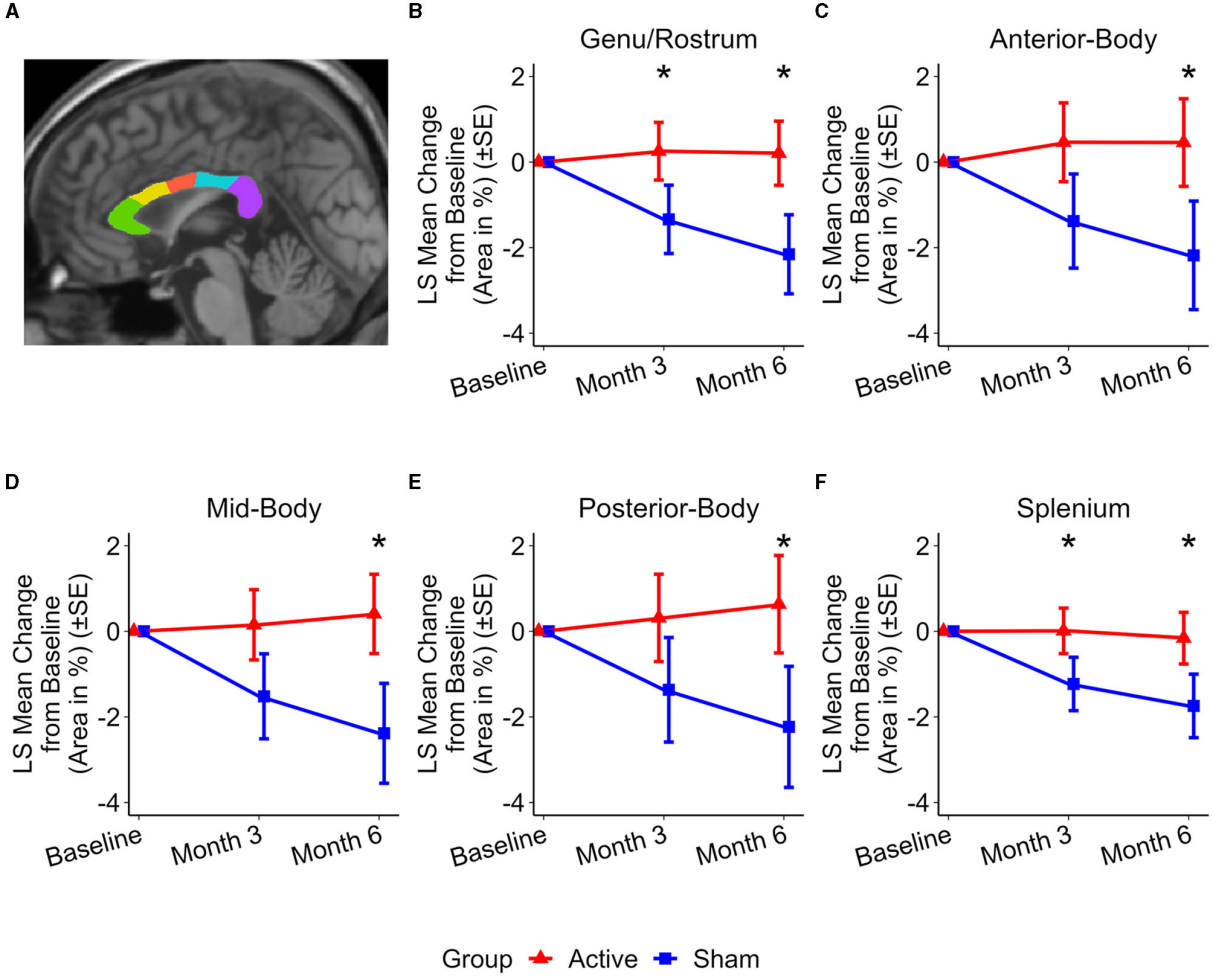
Representative subregional MSP corpus callosum area and group level changes (%) from baseline to 6 months. **(A)** Subregional MSP corpus callosum segmentation from a single participant: genu/rostrum (green), anterior body (yellow), mid body (red), posterior body (blue), and splenium (purple). **(B–F)** Least Squares (LS) Mean changes in subregional corpus callosum area indicate significant differences between the active (red) and sham (blue) treatment groups in favor of the active treatment group. **(B)** Genu/Rostrum (p < 0.02), **(C)** Anterior-body (p < 0.04), **(D)** Mid-body (p < 0.03), **(E)** Posterior-body (p < 0.05), and **(F)** Splenium (p < 0.04). LS Mean area changes at month 3 are significant were observed for **(B)** Genu/Rostrum (p < 0.03) and **(F)** splenium (p < 0.03). Error bars indicate standard error (SE). *p < 0.05.

Figure 2 illustrates the changes in the areas of the corpus callosum subregions. At the 3-month assessment, preservation of area in the anterior and posterior subregions of the corpus callosum was observed in the active treatment group. This preservation is reflected by the following differences in area change compared to the sham group: Genu/Rostrum (1.59 ± 0.70%, *p* < 0.03) and Splenium (1.24 ± 0.54%, *p* < 0.03). At 6 months, the active treatment group exhibited preservation or reduced atrophy in all studied subregions whereas the sham group showed atrophy in all subregions. The differences between the two groups were significant: Genu/Rostrum (2.36 ± 0.90%, *p* < 0.02), Anterior-Body (2.64 ± 1.26%, *p* < 0.04), Mid-Body (2.79 ± 1.18%, *p* < 0.03), Posterior-Body (2.87 ± 1.41%, *p* < 0.05), and Splenium (1.58 ± 0.73%, *p* < 0.04).

### Annual atrophy rates in the corpus callosum

Several longitudinal studies have documented annual reductions in the corpus callosum area in patients with AD, particularly affecting the genu-rostrum and splenium regions (4, 20–22). These are the same regions where we observed the effects of the treatment within 3 months. We compared the one-year projected atrophy rates of the corpus callosum area of participants in the OVERTURE study with those reported for people with and without AD in the literature. Our findings consistently demonstrate either preservation of the corpus callosum area (total, genu/rostrum, anterior, mid, and posterior body) or reduced atrophy (splenium) compared to the rates reported in the literature (Table 3). Observed rates of corpus callosum reduction in the sham group in OVERTURE were in line with previously reported rates of atrophy in people with AD.

**Table 3:** Comparison of corpus callosum changes in literature.

**REFERENCE**	**ESTIMATED CHANGE (per year)**	**POPULATION**	***N* (M/F)**	**AGE mean ±SD (range)**	**MMSE mean ±SD (range)**	**TOTAL CORPUS CALLOSUM**	**GENU ROSTRUM**	**ANTERIOR BODY**	**MID BODY**	**POSTERIOR BODY**	**SPLENIUM**
OVERTURE^T^	%	Sham	17 (10/7)	75.06 ± 11.26 (51–91)	18.88 ± 3.33 (14–25)	−3.46 ± 1.39	−3.68 ± 1.45	−3.68 ± 1.99	−4 ± 1.89	−3.85 ± 2.23	−2.7 ± 1.23
		Active	33 (10/23)	69.21 ± 7.54 (56–79)	21.15 ± 3.45 (14–26)	0.22 ± 1.03	0.12 ± 1.09	0.44 ± 1.48	0.88 ± 1.38	1.23 ± 1.66	−0.48 ± 0.91
Elahi et al. (21)^†^	%	MCI-NC	57 (46/11)	74.1 ± 7.4 (n.a.)	27.7 ± 1.7 (n.a.)	−0.64 ± 0.29 (M only) 0.02 ± 0.60 (F only)	−0.98 ± 0.59 (M only) −0.02 ± 1.20 (F only)	−0.73 ± 0.63	−0.43 ± 0.62	−0.02 ± 0.69	−0.41 ± 0.47 (M only) 0.02 ± 0.96 (F only)
			MCI-C	81 (51/30)	74.1 ± 6.9 (n.a.)	26.7 ± 1.8 (n.a.)	−0.75 ± 0.28 (M only) −1.22 ± 0.36 (F only)				−1.23 ± 0.56 (M only) −2.03 ± 0.73 (F only)	−0.59 ± 0.53	0.09 ± 0.52	0.04 ± 0.58	−0.82 ± 0.44 (M only) −1.75 ± 0.58 (F only)
Teipel et al. (4)^¶^	%			NC	10 (6/4)	65.4 ± 7.2 (52–76)	29.8 ± 0.4 (29–30)				−0.9 ± 5.0				−1.6 ± 6.3	3.9 ± 12.1	−1.6 ± 9.5	−3.7 ± 12.7	0.7 ± 3.6
		AD		21 (11/10)	69.2 ± 8.2 (54–87)	17.4 ± 6.7 (1–28)	−7.7 ± 6.7	−12.1 ± 15.1	−10.3 ± 18.8	−3.0 ± 10.6	4.0 ± 18.1				−7.3 ± 9.3
Zhu et al. (22)^¶^	%	NC	72 (22/50)	75.43 ± 8.23 (n.a.)	29.19 ± 0.85 (n.a.)	−1.69 ± 1.44	n.a.	n.a.	n.a.	n.a.	n.a.
		Decliner	14 (4/10)	77.07 ± 7.69 (n.a.)	29.36 ± 0.93 (n.a.)	−1.37 ± 0.79	n.a.	n.a.	n.a.	n.a.	n.a.
		AD	51 (30/21)	74.96 ± 6.23 (n.a.)	25.92 ± 3.09 (n.a.)	−3.86 ± 2.81	n.a.	n.a.	n.a.	n.a.	n.a.
Bachman et al. (20)^†^	mm^2^	NC	75 (21/54)	75.5 n.a. (60–93)	29.2 n.a. (26–30)	−1.50 ± 1.56	−0.81 ± 0.71	−0.52 ± 0.39	−0.09 ± 0.40	−0.02 ± 0.50	−0.25 ± 0.51
		AD-VM	51 (29/22)	75.7 n.a. (61–90)	26.9 n.a. (17–30)	−3.20 ± 1.86	−1.61 ± 0.84	−0.43 ± 0.45	−0.13 ± 0.46	0.02 ± 0.60	−0.95 ± 0.59
		AD-M	21 (11/10)	73.4 n.a. (64–83)	23.5 n.a. (19–30)	−4.07 ± 3.54	−2.04 ± 1.70	0.36 ± 1.00	0.22 ± 1.03	0.26 ± 1.08	−2.91 ± 1.31

NC, normal controls; AD, Alzheimer's disease; AD-VM, very mild Alzheimer's disease; AD-M, mild Alzheimer's disease; MCI-C, mild cognitive impairment converted to Alzheimer's disease; MCI-NC, mild cognitive impairment NOT converted to Alzheimer's disease; M, male; F, female.

^†^mean ± 95% CI.

^¶^mean ± SD.

^T^LS_mean ± SE (1-year projection).

n.a., data not available.

## Discussion

We assessed the effects of 40Hz gamma stimulation on the area of the corpus callosum in individuals with clinical MCI and AD. Our findings indicate that after 6 months, participants receiving active treatment exhibited either preservation or a reduction in atrophy of the corpus callosum compared to those receiving sham treatment. These results constitute an extension of our previous findings that demonstrated that 40Hz simultaneous audio-visual sensory stimulation preserves white matter integrity (3). The beneficial effects of 40Hz gamma stimulation may be mediated by stimulation-evoked neuronal oscillatory activity, which potentially enhances white matter plasticity, promoting oligodendrogenesis and adaptive myelination (23, 24).

Accelerated loss of corpus callosum area in AD patients is well documented and considered to indicate either axonal loss or loss of myelination of existing interhemispheric axons (25, 26). Brain atrophy reflects neurodegeneration, but the underlying mechanisms that promote atrophy are not fully understood. Intracellular accumulation of toxic hyperphosphorylated tau likely contributes to neurodegeneration in AD. Hyperphosphorylated tau accumulates in axons and disrupts axonal transport (27), perhaps accounting for the white matter volume loss that is present at a very early stage of AD progression (28).

Preclinical studies demonstrated a protective effect of 40Hz steady-state oscillation, due to optogenetic or non-invasive sensory stimulation. Reduced neurodegeneration and brain volume loss are observed in AD-related transgenic mice when steady-state gamma oscillations are evoked over several weeks due to upregulation of cytoprotective proteins and reduced DNA damage (13). Furthermore, in ischemic stroke models, there are also neuroprotective effects of gamma oscillation evoked by either optogenetic (29) or sensory stimulations (30). Recent clinical findings revealed protective effects of sensory-evoked gamma oscillation on brain atrophy in AD patients (2). Evoked gamma oscillation reduced hippocampal volume loss after 3 months of treatment (31) and whole brain volume loss after 6 months of treatment (2). Notably, attenuated cortical thinning of the occipital cortex was also observed, a region that has profound steady state oscillation in response to visual stimulation (32). Reduced neuronal loss due to evoked gamma oscillation may prevent axonal loss as well as decline in corpus callosum structure.

Neuroprotective effects of evoked gamma oscillations on myelin were also observed in preclinical and clinical studies. A recent study reported that sensory-evoked gamma oscillation prevented demyelination in cuprizone multiple sclerosis model by promoting new oligodendrocyte generation and reducing brain inflammation by decreasing levels of proinflammatory molecules (15). These findings are in line with previous results demonstrating that optogenetic activation of neurons promotes oligodendrocyte differentiation and remyelination of axons in the damaged brain tissue by enhancing axon-oligodendroglia interactions (33). Our recent findings showed that 40Hz sensory-evoked gamma oscillation preserved various white matter structures in OVERTURE trial participants (3). Therefore, it is reasonable to consider that preservation of corpus callosum area could be due to both reduced neurodegeneration and demyelination associated with AD progression.

Interhemispheric communication is critical for normal brain function, and there is disrupted connectivity in numerous psychiatric and neurological diseases. Corpus callosum provides the main connection between the brain hemispheres supporting their interhemispheric coordination and communication. In addition to glutamatergic neurons, parvalbumin positive (PV+) GABAergic neurons connect corresponding cortical areas, including visual, auditory, and motor cortices between hemispheres, forming an interhemispheric neuronal network of the cortex (34, 35). PV+ GABAergic neurons play a critical role in generating gamma oscillation (36); we speculate that interhemispheric PV+ GABAergic neurons also contribute to coherent neuronal network gamma activity and interhemispheric synchronization between the two hemispheres. Importantly, PV+ GABAergic neurons take part in evoked steady-state oscillations (37), and their activity could be one of the key mechanisms producing clinical benefits of evoked steady state gamma oscillation in patients. Furthermore, cortical PV+ GABAergic neurons are myelinated by oligodendrocytes and steady state gamma oscillations activate oligodendrocytes and promote myelination (38). Restored myelination of PV+ GABAergic neurons could also contribute to preservation of corpus callosum morphology, potentially improving interhemispheric communication and brain function.

While our study offers novel insights, there are a few limitations, including baseline imbalance in MMSE scores between active and sham groups, short study duration, and small sample size. While baseline scores were adjusted for in our analysis, their variability could still influence neuropathological progression and intervention outcomes. Although no statistical differences were found in baseline corpus callosum area measures, the small sample size may enhance the effects of any baseline discrepancies. Consequently, caution should be used in generalizing our findings, as these imbalances could obscure the true efficacy of the interventions. We also used 1.5T MRI, rather than the more sensitive 3T. Nonetheless, the study's strengths, notably the use of advanced analytical tools and a standardized MRI acquisition protocol may mitigate these limitations to a certain extent. In this study, participants were permitted to adjust the auditory (sound volume) and visual (light intensity) stimulation settings within predefined limits for comfort. Although these adjustments were allowed, they were not included in the statistical analysis, introducing a potential variable that could influence the outcomes. This should also be acknowledged as a limitation of our analysis.

Our study represents a detailed retrospective analysis of Phase 2 data, specifically focusing on identifying critical structural endpoints in the corpus callosum. The study was not designed to correlate these structural changes directly with functional or cognitive outcomes. We anticipate that forthcoming larger-scale studies, such as the Hope pivotal study (*N* > 500), will be better equipped to examine how alterations in the corpus callosum structure may impact cognitive functioning and disease progression in Alzheimer's disease and other neurodegenerative disorders prone to white matter abnormalities.

In conclusion, we demonstrate that 40Hz noninvasive gamma sensory stimulation is a promising intervention to preserve corpus callosum integrity. By comparing our findings with the observed corpus callosum atrophy in AD found in literature, we provide novel insights into possible intervention to preserve corpus callosum integrity. Our research paves the way for future investigations aimed at unraveling the mechanistic underpinnings of these findings. The potential for this therapy to transform the clinical management of AD and possibly other neurodegenerative diseases by possibly aiding to preserve network structure marks an exciting frontier in neuroscience and clinical research.

## Data Availability

The datasets presented in this article are not readily available because the data are not publicly available due to information that could compromise the confidentiality of research participants. Requests to access the datasets should be directed to Ralph Kern, rkern@cognitotx.com.
